# Intramuscular inflammatory and resolving lipid profile responses to an acute bout of resistance exercise in men

**DOI:** 10.14814/phy2.14108

**Published:** 2019-06-30

**Authors:** Luke Vella, James F. Markworth, Michelle M. Farnfield, Krishna R. Maddipati, Aaron P. Russell, David Cameron‐Smith

**Affiliations:** ^1^ Institute for Physical Activity and Nutrition School of Exercise and Nutrition Sciences Deakin University Geelong Victoria Australia; ^2^ Department of Sports Development and Recreation University of Bath Bath United Kingdom; ^3^ Liggins Institute University of Auckland Auckland New Zealand; ^4^ Department of Molecular & Integrative Physiology University of Michigan Ann Arbor Michigan; ^5^ Bioactive Lipids Research Program and Lipidomics Core Department of Pathology School of Medicine Wayne State University Karmanos Cancer Institute Detroit Michigan

**Keywords:** Exercise recovery, inflammation, inflammatory resolution, lipids

## Abstract

Lipid mediators including classical arachidonic acid‐derived eicosanoids (e.g. prostaglandins and leukotrienes) and more recently identified specialized pro‐resolving‐mediator metabolites of the omega‐3 fatty acids play essential roles in initiation, self‐limitation, and active resolution of acute inflammatory responses. In this study, we examined the bioactive lipid mediator profile of human skeletal muscle at rest and following acute resistance exercise. Twelve male subjects completed a single bout of maximal isokinetic unilateral knee extension exercise and muscle biopsies were taken from the *m*.vastus lateralis before and at 2, 4, and 24 h of recovery. Muscle tissue lipid mediator profile was analyzed via liquid chromatography–mass spectrometry (LC‐MS)‐based targeted lipidomics. At 2 h postexercise, there was an increased intramuscular abundance of cyclooxygenase (COX)‐derived thromboxanes (TXB_2_: 3.33 fold) and prostaglandins (PGE_2_: 2.52 fold and PGF_2*α*_: 1.77 fold). Resistance exercise also transiently increased muscle concentrations of lipoxygenase (LOX) pathway‐derived leukotrienes (12‐Oxo LTB_4_: 1.49 fold and 20‐COOH LTB_4_: 2.91 fold), monohydroxy‐eicosatetraenoic acids (5‐HETE: 2.66 fold, 12‐HETE: 2.83 fold, and 15‐HETE: 1.69 fold) and monohydroxy‐docosahexaenoic acids (4‐HDoHE: 1.69 fold, 7‐HDoHE: 1.58 fold and 14‐HDoHE: 2.35 fold). Furthermore, the abundance of CYP pathway‐derived epoxy‐ and dihydroxy‐eicosatrienoic acids was increased in 2 h postexercise biopsies (5,6‐EpETrE: 2.48 fold, 11,12‐DiHETrE: 1.66 fold and 14,15‐DiHETrE: 2.23 fold). These data reveal a range of bioactive lipid mediators as present within human skeletal muscle tissue and demonstrate that acute resistance exercise transiently stimulates the local production of both proinflammatory eicosanoids and pathway markers in specialized proresolving mediator biosynthesis circuits.

## Introduction

Skeletal muscle is a remarkably heterogeneous tissue with the capacity to adapt and respond to external stress. It is well established that intense resistance exercise can lead to improvements in muscle strength through changes in muscle fiber type, myofibrillar hypertrophy, and neuromuscular mechanisms (Flück 2006). However, unaccustomed exercise, especially when comprising a large eccentric component, can cause skeletal muscle injury and initiate an acute inflammatory response (Armstrong et al. 1991; Faulkner et al. 1993; Tidball 1995). Experimental models targeted at manipulating the postexercise inflammatory response have identified that exercise‐induced inflammation is a key regulatory feature in the normal process of tissue regeneration and adaptation following acute muscle damage (Urso 2013; Markworth et al. 2014; Roberts et al. 2015). This suggests that molecular signaling events occurring early during acute inflammation play an active role in promoting the restoration of normal tissue function and promote skeletal muscle adaptation following an exercise stimulus.

The humoral and local muscular changes that occur during exercise‐induced inflammation closely resemble that of an acute phase response to cellular stress. Exercise stimulus triggers the production of proinflammatory signaling molecules, establishing a chemotactic gradient and the diapedesis as well as potential infiltration of inflammatory leukocytes (Paulsen et al. 2012). These chemoattractants consist of lipid‐derived mediators such as leukotrienes (LTs) and prostaglandins (PGs), as well as protein mediators, including cytokines and chemokines (Serhan et al. 2008). The usual outcome of an acute inflammatory response is its successful resolution and repair of damaged tissue (Serhan and Savill 2005). Traditionally, the resolution of inflammation was thought to be a passive process involving the dilution and catabolism of pro‐inflammatory mediators leading to the exodus of leukocytes from the site of muscle damage. However, with the discovery of novel classes of lipid‐derived mediators, the resolution of acute inflammation is now seen as an active and finely controlled biochemical and metabolic process that may provide a critical link between cellular stress and tissue regeneration/adaptation (Levy et al. 2001; Bannenberg et al. 2005).

Lipid mediators are biosynthesized endogenously from essential omega‐6 (n‐6) and omega‐3 (n‐3) polyunsaturated fatty acids (PUFA) and are involved in a wide range of physiological and pathophysiological processes (Bannenberg et al. 2005). The majority of research in postexercise inflammation has focused on classical prostaglandins (synthesized via cyclooxygenase (COX) enzymes, COX‐1 and COX‐2) and leukotrienes (synthesized via the 5‐lipoxygenase (5‐LOX) enzyme), which are derived from the n‐6 PUFA arachidonic acid (AA). These lipid mediators play a diverse role in stimulating acute inflammation by controlling local blood flow, vascular permeability, cytokine production, leukocyte chemotaxis, and sensation of pain (Markworth et al. 2013). On the other hand, a second class of eicosanoids also generated from AA, termed the lipoxins (LX) (Serhan et al. 1995; Maddox and Serhan 1996; Ryan and Godson 2010), together with more recently identified eicosapentaenoic (EPA) (E‐Series) and docosahexaenoic acid (DHA) (D‐Series)‐derived resolvins (Rv), protectins (PD) (Serhan et al. 2000, 2002, 2015; Hong et al. 2003; Serhan 2006; Duvall and Levy 2016), and maresins (MaR) (Serhan et al. 2009 & 2012) have been shown to play proresolution functions following acute inflammation. These novel lipid mediators, collectively termed specialized proresolving mediators (SPMs), act to block acute inflammatory signals by inhibiting proinflammatory cytokine production and subsequent neutrophil chemotaxis (Schwab et al. 2007; Serhan and Petasis 2011). They simultaneously promote the nonphlogistic infiltration of blood monocytes/macrophages and stimulate tissue macrophages to phagocytize and clear apoptotic neutrophils while promoting wound healing (Maddox and Serhan 1996; Godson et al. 2000).

SPMs are formed during inflammatory transcellular interactions, involving the sequential actions of two or more cell types expressing the required LOX and/or COX enzymes in a compartmentalized manner. During the time‐course of inflammation cell–cell interactions between platelets, leukocytes, the vasculature and resident tissue cells facilitates the transcellular biosynthesis of unique SPMs (Markworth et al. 2016b). The temporal regulation of these lipids is therefore specific to the tissue type, inciting inflammatory stimulus (Levy et al. 2001; Bannenberg et al. 2005). For example, LX biosynthesis involves cellular interactions between 5‐LOX expressing neutrophils with 12‐lipoxygenase (12‐LOX) expressing platelets or 15‐lipoxygenase (15‐LOX) expressing M2 monocytes (Serhan et al. 1984). Recent findings from Markworth et al. (2013) demonstrated that SPMs, including lipoxins, resolvins, and protectins, were elevated in human blood serum samples collected following an acute bout of resistance exercise. Peak induction of proinflammatory mediators including the prostaglandins and leukotrienes occurred during the early stages of postexercise muscle recovery (1–2 h), while elevated concentrations of specific SPMs were detected during both early (0–3 h: LXA_4_/LXB_4_, RvE1 and RvD1) and later (24 h: PD1) stages of muscle recovery. In this study, we used the same targeted lipidomics approach to characterize the time‐course of changes in concentrations of eicosanoid and docosanoid species locally within human skeletal muscle tissue following an acute bout of resistance exercise. We aimed to identify which species of bioactive lipid mediators are present within skeletal muscle tissue and hence may be locally generated and acting following an acute bout of resistance exercise. We hypothesized that there would be a rapid increase in pro‐inflammatory prostaglandin and leukotriene biosynthesis, followed by the activation of SPM pathways at the onset of inflammatory resolution. Identification of the lipid mediator profile of skeletal muscle and ability of exercise stress to modulate intramuscular bioactive lipids will help to contribute to the understanding of a biologically active inflammatory resolution pathway that may be essential to muscle recovery and adaptation following an inflammatory event.

## Materials and Methods

### Subjects

As previously described (Farnfield et al. 2009), fourteen untrained but recreationally active men aged 18–25 years were recruited to participate in the acute exercise study. A subset of 12 male participants, for which sufficient muscle biopsy tissue remained, were included in the analysis performed here (Table 1). Exclusion criteria included participation in regular resistance exercise within one year prior to commencing the study, and/or the consumption of any nutritional or purported muscle‐building supplements. Each participant also completed a medical history questionnaire to identify any potential risk factors that would prevent the subjects from completing strenuous exercise.

**Table 1:** Subject characteristics. Values are mean ± SEM of n = 12 participants

Age (y)	Height (cm)	Body mass (kg)	BMI (kg.m^2^)
22.1 ± 0.6	178.8 ± 2.2	74.6 ± 2.6	23.3 ± 0.7

### Ethics approval

Each participant was provided with a written and oral explanation of the nature of the study and potential risks of the experimental procedures before providing written consent to participate. All procedures involved in the study were formally approved by the Deakin University Human Research Ethics Committee (DUHREC 2004‐017) and muscle biopsy procedures were performed in order with Helsinki declaration.

### Familiarization

At least 7 days prior to the trial day, each subject completed a familiarization session on the Cybex NORM dynamometer (Cybex International Inc. UK). The session involved performing isokinetic maximal voluntary contractions (iMVC) during concentric and eccentric knee extension exercise. Maximal force production measured as peak torque (N.m) was determined at 60°/s over 12 maximal concentric and eccentric contractions. Subjects were provided with verbal encouragement throughout the test to ensure maximal effort.

### Experimental design

On the morning of the trial, subjects reported to the laboratory in an overnight fasted state having abstained from alcohol, caffeine, and tobacco for the previous 24 h. Participants rested in a supine position for 30 min, following which a resting muscle biopsy sample was collected. Each participant then completed an acute bout of maximal concentric and eccentric isokinetic unilateral knee extension exercise on the Cybex NORM dynamometer. Subjects completed three sets of 12 maximal voluntary repetitions at a constant speed of 60°/s with 2 min of rest between each set. Subjects were instructed to contract maximally during each repetition and were provided with verbal encouragement throughout each set. Further muscle biopsy samples were obtained from the exercised leg at 2 and 4 h after completion of the exercise protocol. The following morning, subjects reported to the laboratory again in an overnight fasted state for a final follow up 24 h postexercise muscle biopsy sample.

### Muscle biopsy procedure

Muscle biopsy samples were obtained from the *m*.vastus lateralis under local anesthesia (Xylocaine 1%) using a percutaneous needle biopsy technique modified to include suction (Buford et al. 2009). A section of excised tissue was rapidly snap frozen in liquid nitrogen and stored at −80°C for further analysis. Repeat muscle biopsy samples were collected from the same leg through separate incisions separated by at least 2 cm from the previous biopsy site to minimize the risk of any localized inflammation arising from the biopsy procedure confounding exercise‐induced inflammation.

### Liquid chromatography‐mass spectrometry (LC‐MS)

Muscle biopsy samples were weighed and homogenized in 1 mL phosphate buffered saline (50 mmol/L phosphate containing 0.9% sodium chloride, pH 7.4), using Zirconium beads on a high‐frequency oscillator (Precellys homogenizer, Bertig Instruments). The homogenates were centrifuged at 6000 *g* for 10 min and the supernatant was collected for the extraction of fatty acyl lipid mediators using C18 solid phase extraction cartridges as described earlier (Markworth et al. 2013; Maddipati et al. 2014, 2016). Fatty acyl lipid mediator extracts were subjected to LC‐MS analysis essentially as described before (Markworth et al. 2013, 2016a). Under the LC‐MS conditions employed, the detection limit for most of the lipid mediators was 1 pg on the column and the quantitation limit was 5 pg on the column with a signal‐to‐noise ratio > 3. Tissue weights from each sample (range: 14–70 mg, average: 43 mg, inter‐quartile range: 32–56 mg) were used for normalization of the LC‐MS data and the data are reported as ng per gram (ng/g) of tissue.

### Statistics

Statistical analysis was performed using SigmaPlot v12.3 (Systat Software Inc, Chicago, IL). Data were analyzed using a one‐way repeated measures ANOVA. Following a statistically significant main ANOVA effect, Student–Newman–Keuls post hoc tests were used to determine the significance of pair‐wise comparisons between individual time points. Data is presented as mean ± standard error of the mean (SEM). Statistical significance was set at *P < *0.05.

## Results

### Metabolipidomic profile of human skeletal muscle tissue

Lipid mediator profiles of human skeletal muscle biopsies were generated via targeted LC‐MS/MS based metabolipidomics. Of the total 125 multiple reaction monitoring (MRM) transitions, 84 unique lipid mediators were reliably detected in resting skeletal muscle tissue (signal/noise ratio > 3 in ≥ 50% of samples) (Table S1). Detected analytes included a range n‐6 and n‐3 PUFA metabolites enzymatically derived from the COX, LOX, and CYP pathways (Table S1). Metabolites of linoleic acid (LA, 18:2n‐6) including hydroxy‐octadecadienoic acid (9‐, 13‐HODEs) and epoxy‐octadecadienoic acids (9(10)‐, 12(13)‐EpOMEs) were most abundant (50–100 ng/g), followed by major enzymatic metabolites of the n‐6 PUFA AA (20:4n‐6). Numerous metabolites of the n‐3 PUFAs EPA (20:5n‐3) and DHA (22:6n‐3) were also detected at relatively lower concentrations.

### Cyclooxygenase pathways

#### Omega‐6 derived

**Figure 1:**
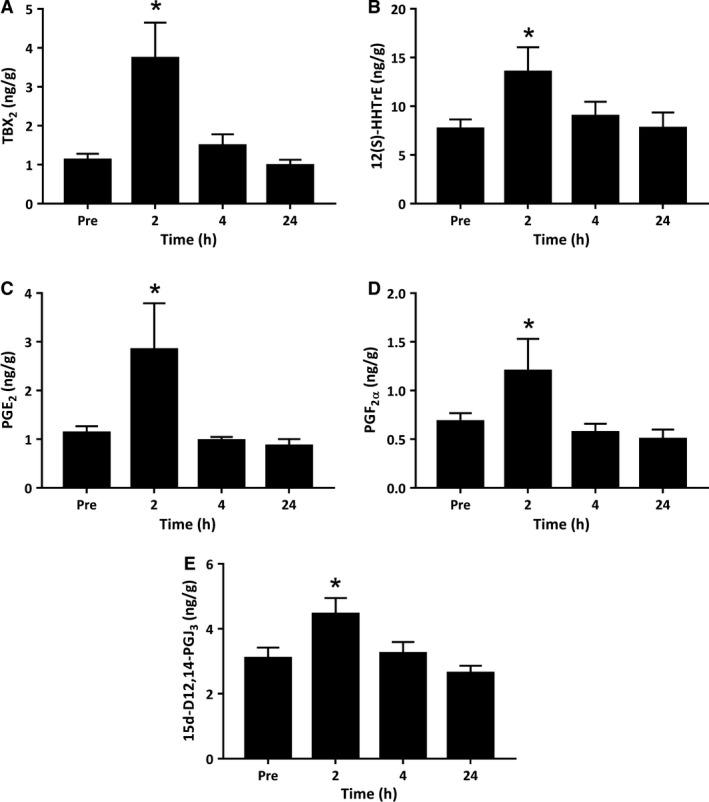
Metabolites of the cyclooxygenase (COX) pathway derived from arachidonic acid and eicosapentaenoic acid. Values depicted are mean values ± SEM. * denotes statistical significance compared to pre‐exercise values (*P *<* *0.05).

COX enzymes catalyze the first step in the conversion of AA to prostaglandins (PGE_2_, PGF_2ɑ_, PGD_2_, and PGI_2_) and thromboxane (TXA_2_). TXA_2_ is highly unstable and nonenzymatically decomposes to TXB_2_ and 12(S)‐HHTrE. Thus, both these metabolites serve as surrogate markers of TXA_2_ biosynthesis. TXB_2_ and 12(S) HHTrE were both detected within resting skeletal muscle tissue at concentrations of 1.12 ng/g and 7.68 ng/g, respectively (Fig. 1). At 2 h postexercise, muscle TXB_2_ increased to 3.73 ng/g (*P *=* *0.002) (Fig. 1A) and 12(S)‐HHTrE increased to 13.50 ng/g (*P *=* *0.002) (Fig. 1B)_._ The prostaglandins PGE_2_ and PGF_2ɑ_ were also detected in resting skeletal muscle tissue at concentrations of 1.13 ng/g and 0.68 ng/g, respectively. At 2 h postexercise, intramuscular PGE_2_ increased to 2.84 ng/g (*P = *0.009) (Fig. 1C) and PGF_2ɑ_ increased to 1.20 ng/g (*P *=* *0.013) (Fig. 1D). Intramuscular TXB_2_, 12(S)HHTrE, PGE_2_ and PGF_2*α*_ were no longer elevated above preexercise levels by 4 h and 24 h of recovery from the exercise bout. Other major AA‐derived prostaglandins including PGD_2_ and PGI_2_ (measured as the stable downstream nonenzymatic metabolite 6‐keto‐PGF_1*α*_) were below the limit of detection of the assay used here in human muscle biopsy samples collected both before and after the resistance exercise intervention (Table S1).

#### Omega‐3 derived

The majority of 3‐series (EPA derived) prostaglandins were below the limit of detection of our assay in human skeletal muscle tissue (Table S1). However, a downstream bioactive metabolite of the EPA‐derived PGD_3_, 15‐Deoxy‐Δ^12,14^‐prostaglandin J_3_, was detected at concentrations of 3.09 ng/g in resting muscle. Furthermore, 15‐Deoxy‐Δ^12,14^‐prostaglandin J_3_ increased 1.35 fold to concentrations of 4.45 ng/g at 2 h postexercise (*P = *0.010) (Fig. 1E). 15‐Deoxy‐Δ^12,14^‐prostaglandin J_3_ returned to basal levels by 4 h (*P = *0.764) and 24 h (*P = *0.344).

### Lipoxygenase pathways

#### 5‐LOX

**Figure 2:**
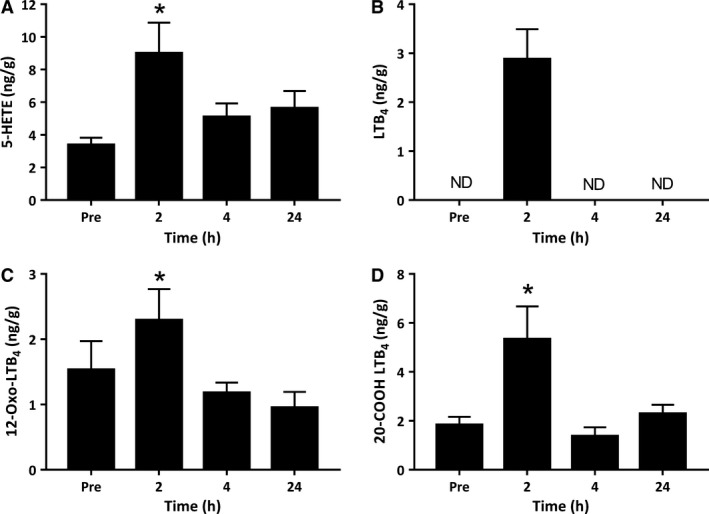
Metabolites of the 5‐lipoxygenase (5‐LOX) pathway derived from arachidonic acid. Values depicted are mean values ± SEM. * denotes statistical significance compared to preexercise values (*P *<* *0.05).

The 5‐LOX pathway primarily converts AA substrate to 5‐hydroperoxy‐eicosatetranoic acid (5‐HpETE), which can be reduced to 5‐hydroxy‐eicosatetranoic acid (5‐HETE), or undergo further metabolism via 5‐LOX to form the leukotrienes. 5‐HETE was detected in resting muscle at a concentration of 3.38 ng/g (Fig. 2A). Muscle 5‐HETE levels increased at 2 h postexercise to 8.99 ng/g (p = 0.017) (Fig. 2A). Leukotriene B_4_ (LTB_4_) was below the level of detection of our assay in the majority of resting muscle biopsies, but was consistently detected at concentrations of ~3 ng/g at 2 h of recovery from the bout of resistance exercise (Fig. 2B). Furthermore, downstream degradation products of LTB_4_, including 12‐Oxo‐LTB_4_ and 20‐COOH‐LTB_4_ were both detected in resting muscle and increased in abundance at 2 h postexercise to concentrations of 2.29 ng/g (*P *<* *0.041) (Fig. 2C) and 5.33 ng/g (*P *<* *0.001) (Fig. 2D), respectively. Intramuscular 5‐LOX products of AA including 5‐HETE, 12‐Oxo‐LTB_4_ and 20‐COOH‐LTB_4_ were no longer increased above basal levels at 4 h or 24 h of recovery.

#### 12‐LOX

**Figure 3:**
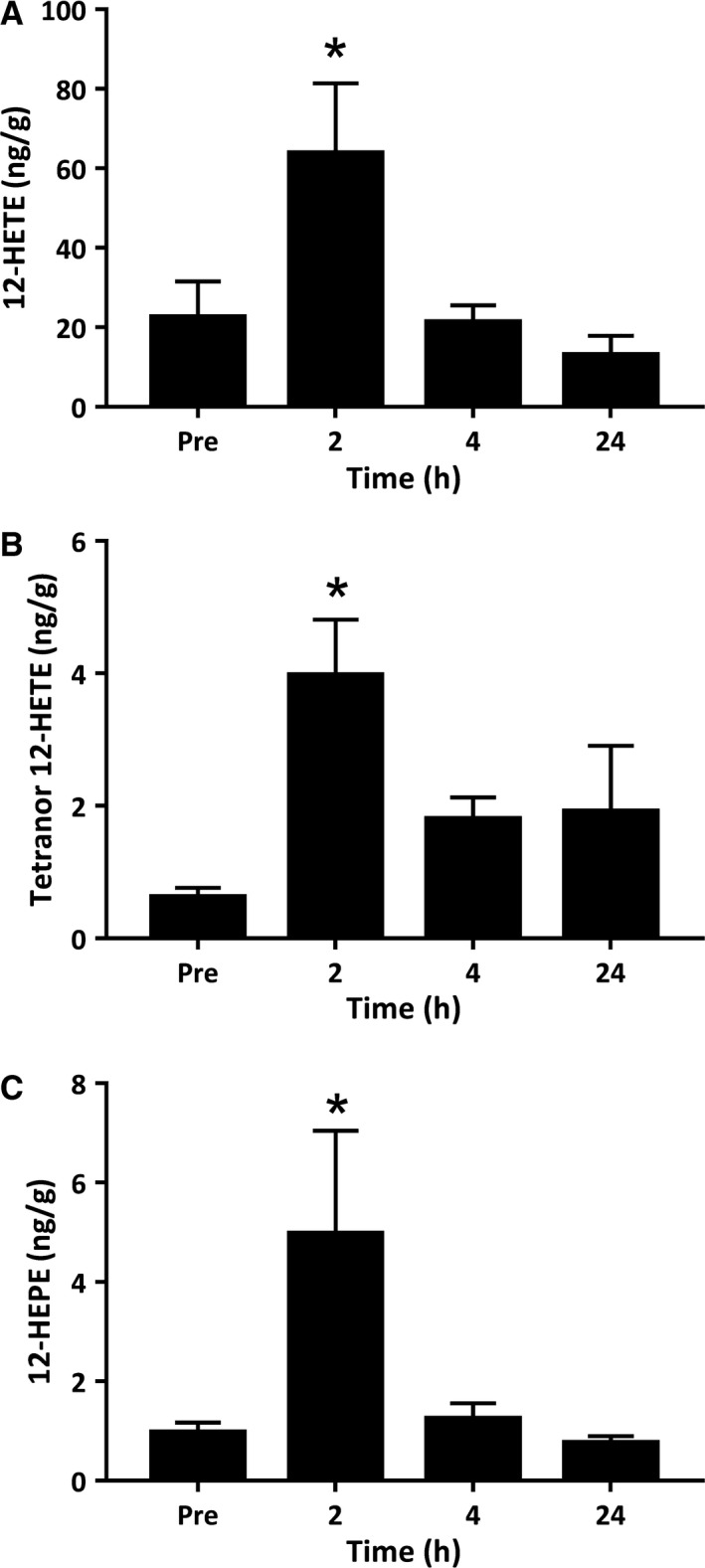
Metabolites of the 12‐lipoxygenase (12‐LOX) pathway derived from arachidonic acid and eicosapentaenoic acid. Values depicted are mean values ± SEM. * denotes statistical significance compared to preexercise values (*P *<* *0.05).

The 12‐LOX enzyme is expressed primarily in human platelets and metabolizes AA to form 12‐hydroxy‐eicosatetraenoic acid (12‐HETE), which is a key stimulator of leukocyte chemotaxis and platelet aggregation (Dobrian et al. 2011; Yeung and Holinstat 2011; Yin et al. 2011). 12‐HETE was by far the most abundant monohydroxylated‐FA product detected in resting muscle tissue, present at concentrations of 22.51 ng/g. Muscle 12‐HETE further increased above resting levels at 2 h postexercise to concentrations of 63.81 ng/g (*P *=* *0.01) (Fig. 3A). Tetranor 12‐HETE, a downstream degradation product of 12‐HETE, was also detected in resting muscle tissue at lower concentrations of 0.62 ng/g and increased markedly at 2 h postexercise to reach intramuscular concentrations of 3.97 ng/g (*P *=* *0.006) (Fig. 3B). Furthermore, the 12‐LOX metabolites of n‐3 EPA, 12‐hydroxy‐eicosapentaenoic acid (12‐HEPE) was present in resting muscle biopsies and increased at 2 h postexercise (*P *=* *0.016) (Fig. 3C). By 4 h and 24 h of recovery, intramuscular 12‐LOX products including 12‐HETE, tetranor 12‐HETE, and 12‐HEPE no longer differed from resting levels.

#### 15‐LOX

**Figure 4:**
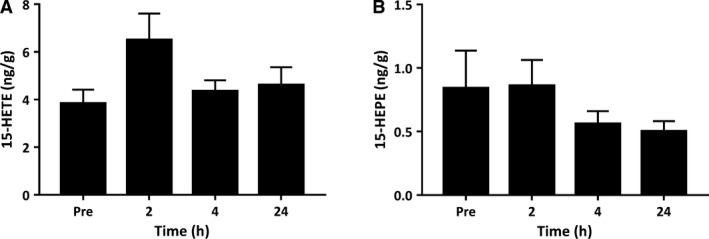
Metabolites of the 15‐lipoxygenase (15‐LOX) pathway derived from arachidonic acid and eicosapentaenoic acid. Values depicted are mean values ± SEM. * denotes statistical significance compared to preexercise values (*P *<* *0.05).

The 15‐LOX pathway converts AA to 15‐hydroxy‐eicosatetranoic acid (15‐HETE) (Wuest et al. 2012). 15‐HETE was detected in resting muscle at 3.84 ng/g. Muscle 15‐HETE tended to increase at 2 h postexercise to 6.50 ng/g, but this did not achieve statistical significance (*P *=* *0.10) (Fig. 4A). The 15‐LOX metabolite of n‐3 EPA, 15‐hydroxy‐eicosapentaenoic acid (15‐HEPE) was also detected in resting muscle tissue, but was not influenced by the exercise intervention (main effect *P* = 0.308) (Fig. 4B).

#### Docosanoids

In addition to the 20‐carbon PUFA AA and EPA, LOX pathway converts the 22‐carbon n‐3 PUFA DHA to docosanoid metabolites which most notably are key pathway markers and intermediates in the biosynthesis of the SPM family of bioactive lipid mediators.

**Figure 5:**
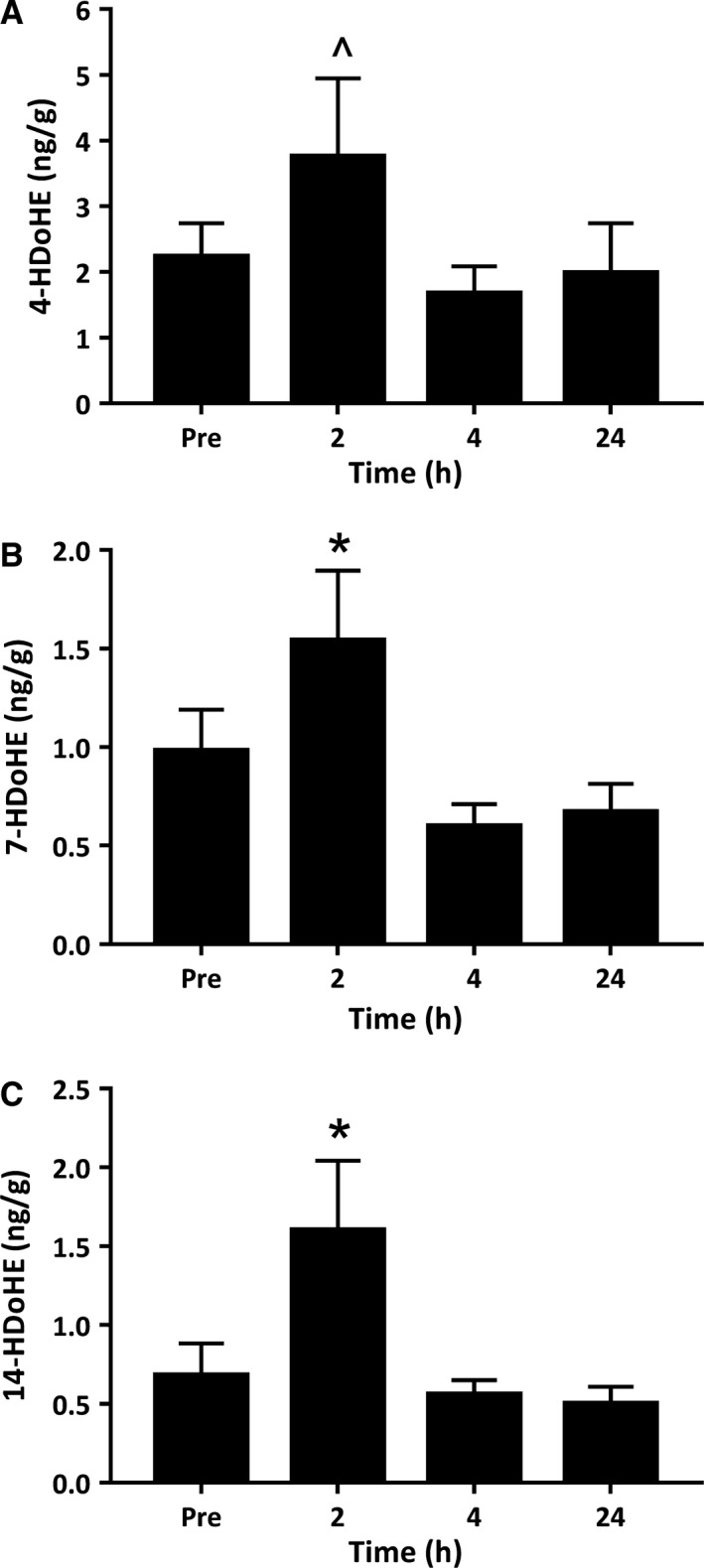
Metabolites of the lipoxygenase pathways derived from docosahexaenoic acid. Values depicted are mean values ± SEM. * denotes statistical significance compared to pre‐exercise values (*P *<* *0.05). ^ denotes statistical significance compared to 4 and 24 h postexercise values (*P *<* *0.05).

The 5‐LOX enzyme oxidizes n‐3 DHA to form the monohydroxylated‐DHA (HDoHE) products 4‐hydroxy‐docosahexanoic acid (4‐HDoHE) and 7‐hydroxy‐docosahexanoic acid (7‐HDoHE). Both 4‐ and 7‐HDoHE were detected in resting muscle at concentrations of 2.23 ng/g (Fig. 5A) and 0.98 ng/g (Fig. 5B), respectively. 7‐HDoHE increased at 2 h postexercise to 1.54 ng/g (*P *=* *0.008) (Fig. 5B). Similarly, muscle 4‐HDoHE tended to increase from preexercise levels at 2 h postexercise (*P *=* *0.069), and was statistically greater at 2 h compared than both 4 h (*P *=* *0.011) and 24 h (*P *=* *0.016) of recovery (Fig. 5A).

The 12‐LOX enzyme converts the 22‐carbon DHA to 14‐hydroperxy‐docosahexanoic acid (14‐HpDoHE), which can then be reduced to 14‐HDoHE or metabolized to form the maresins (MaR 1 & 2) via the further action of 12‐LOX. Similarly, the 15‐LOX enzyme converts DHA to 17‐hydroxy‐docosahexanoic acid (17‐HDoHE) which can be converted into the D‐series resolvins via the subsequent action of 5‐LOX. Therefore, 14‐HDoHE and 17‐HDoHE are pathway markers of increased MaR and RvD biosynthesis, respectively. We detected 14‐HDoHE in resting skeletal muscle at a concentration of 0.68 ng/g (Fig. 5C). Muscle 14‐HDoHE increased at 2 h postexercise to concentrations of 1.60 ng/g (*P *=* *0.005) (Fig. 5C). In contrast, 17‐HDoHE was not found to be present at detectable concentrations within human muscle biopsies at rest or at any time‐point throughout exercise recovery (Table S1).

Multiple reaction monitoring (MRM) transitions corresponding to mature SPMs including the lipoxins (LXA_4_, LXB_4,_ LXA_5_), E‐series resolvins (RvE1 & RvE3), D‐series resolvins (RvD1, RvD2, RvD5, RvD6), protectins (PD1 & 10S,17S‐DiHDoHE) and maresins (MaR1) were additionally monitored by our LC‐MS/MS assay. Resting muscle tissue was found to contain low but detectable concentrations of RvD6 (1.04 ng/g), PD1 (0.64 ng/g) and MaR1 (0.75 ng/g). The greatest average intramuscular concentrations of RvD6 (1.81 ng/g), PD1 (2.58 ng/g), and MaR1 (1.33 ng/g) were observed at 2 h postexercise. Despite this, the low and sporadic concentrations in certain subjects at particular time‐points, combined with our repeated measures study design, precluded statistical analysis. Other mature SPMs including LXA_4_, LXB_4_, LXA_5_, RvD1, RvE3, RvD1, RvD2, RvD5 and the protectin D1 isomer 10S,17S‐DiHDoHE were not found to be present at detectable levels locally within human muscle tissue biopsies at rest or throughout 24 h of postexercise recovery under the conditions used here (Table S1).

#### Epoxygenase pathway

**Figure 6:**
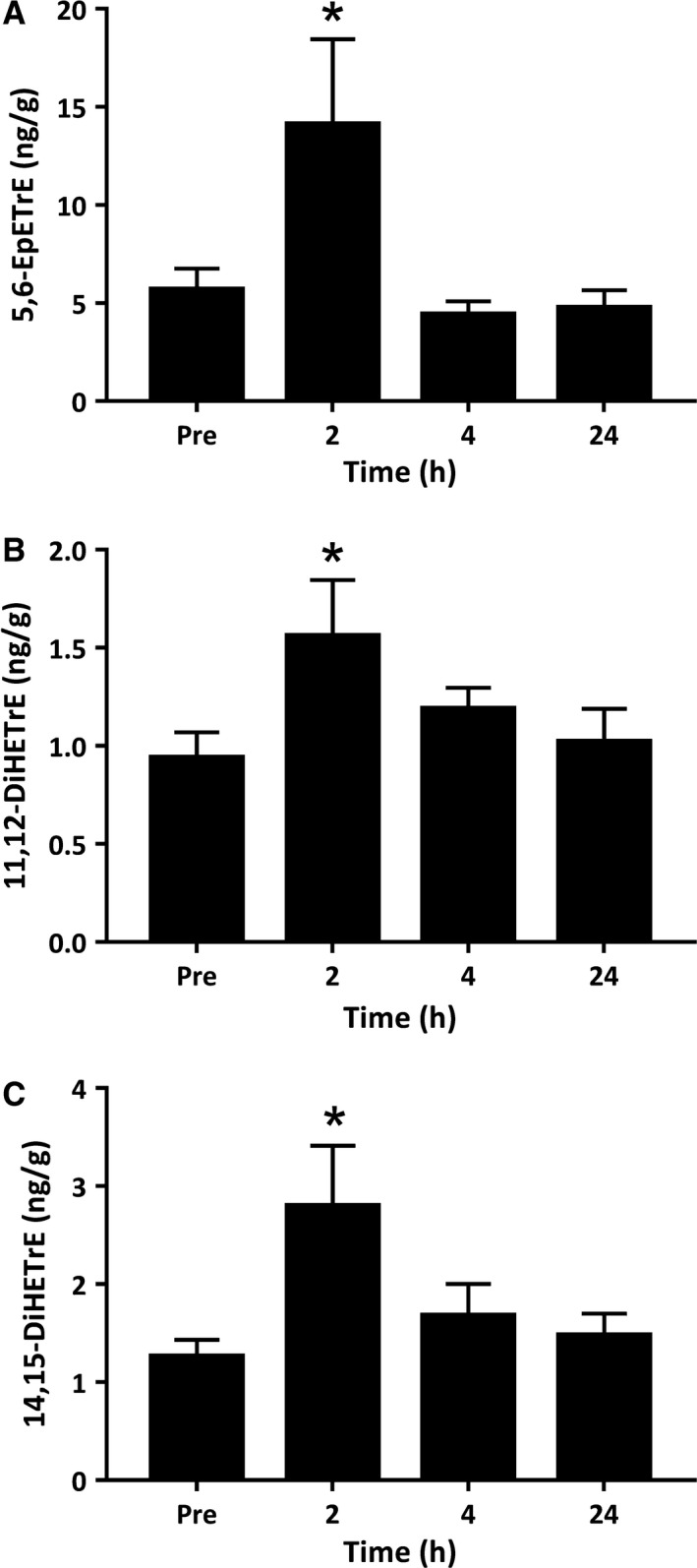
Metabolites of the epoxygenase pathway derived from arachidonic acid. Values depicted are mean values ± SEM. * denotes statistical significance compared to preexercise values (*P *<* *0.05).

The cytochrome P‐450 (CYP) enzymes metabolize n‐6 PUFA AA to a family of epoxyeicosatrienoic acid (EpETrE) regioisomers. Once formed, these bioactive EpETrEs are rapidly metabolized by the soluble epoxide hydrolase (sEH) enzyme to form corresponding downstream dihydroxyeicosatrienoic acids (DiHETrEs) vicinal diols. AA epoxides including 5,6‐, 8,9‐, 11,12‐ and 13,14‐EpETrE, were detected in resting muscle tissue at concentrations between 3 and 10 ng/g. Despite this, 5,6‐EpETrE was the only primary enzymatic epoxy AA‐metabolite that showed an effect for the exercise intervention, increasing threefold to 14.12 ng/g at 2 h postexercise (*P *<* *0.003) (Fig. 6A). Downstream epoxide products of the sEH enzyme, 11,12‐DiHETrE and 14,15‐DiHETrE were present at much lower absolute concentrations in resting muscle (~1 ng/g), but increased in response to exercise with a peak response at 2 h of recovery (11,12‐DiHETrE *P *=* *0.026, 14,15‐DiHETrE *P *=* *0.011) (Fig. 6B and C). The 5,6‐DiHETrE regioisomer was also detected at low levels in resting muscle, but was unchanged during postexercise recovery (Table S1).

**Figure 7:**
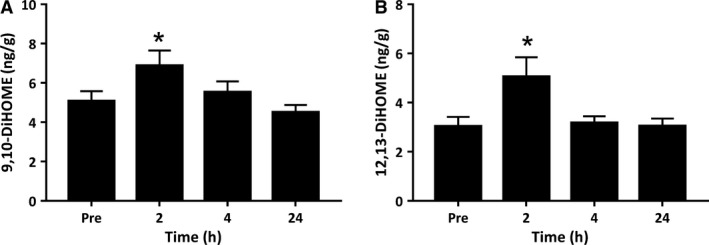
Metabolites of the epoxygenase pathway derived from linoleic acid. Values depicted are mean values ± SEM. * denotes statistical significance compared to preexercise values (*P *<* *0.05).

CYP epoxidase enzymes also have the capacity to metabolize the n‐6 linoleic acid to bioactive lipid mediators including 9(10)‐EpOME and 12(13)‐EpOME and their downstream sEH products 9(10)‐DiHOME and 12(13)‐DiHOME. Both 9(10)‐ and 12(13)‐EpOME were highly abundant in resting human muscle present at concentrations of 100.87 ng/g and 54.36 ng/g, respectively, but were not influenced by the exercise intervention (Table S1). In contrast, 9(10)‐ and 12(13)‐DiHOME were present at lower concentrations in resting muscle, but increased significantly at 2 h postexercise (*P* = 0.034 and *P* = 0.020 respectively) (Fig. 7). CYP pathway metabolites of both AA and LA no longer differed from preexercise levels by 4‐ and 24‐h of postexercise recovery.

## Discussion

The study explored the intramuscular lipid mediator response following an acute bout of resistance exercise. We identified 84 unique lipid mediators as present within skeletal muscle tissue. The early postexercise response was characterized by increased tissue concentrations of a range of bioactive lipid derivatives of the cyclooxygenase (COX‐1 & 2), lipoxygenase (5‐, 12‐ & 15‐LOX), and epoxygenase (CYP) pathways. Unexpectedly, however, peak induction of both proinflammatory and SPM pathway intermediates occurred simultaneously at 2 h of recovery. The findings from this study identify a complex parallel adaptive lipid response to resistance exercise that may play an essential role in regulating the onset and resolution of acute exercise‐induced skeletal muscle inflammation.

This is the first paper to use a targeted lipidomics approach to extensively characterize the lipid mediator profile of human skeletal muscle tissue at rest and in response to acute exercise. The majority of human research exploring the effect of exercise on lipid species has focused primarily on a select few prostaglandins, specifically PGE_2_ and PGF_2ɑ_, likely due to their complex role in regulating acute inflammation, perceptions of pain and purported roles in muscle cell growth/regeneration (Karamouzis et al. 2001a, 2001b; Trappe et al. 2001, 2006). Although some studies have reported elevated circulating levels of PGE_2_ following exercise‐induced muscle injury (Dousset et al. 2007; Uchida et al. 2009; Tartibian et al. 2011; Markworth et al. 2013), these findings have not yet been replicated in skeletal muscle tissue (Trappe et al. 2001; Paulsen et al. 2010). PGF_2ɑ_ increases locally within skeletal muscle tissue following both eccentric (Trappe et al. 2001) and isotonic resistance exercise protocols (Trappe et al. 2006). It plays a significant role in *in vitro* myofiber hypertrophy (Markworth and Cameron‐Smith 2011) and postexercise muscle protein synthesis (Trappe et al. 2002). We previously found a significant increase in serum levels of the circulating PGF_2ɑ_ metabolite 15‐keto‐PGF_2ɑ_ early (1 h) following resistance exercise in humans (Markworth et al. 2013). In this study, PGE_2_ and PGF_2ɑ_ were the most abundant AA‐derived PGs detected within muscle tissue and both transiently increased in abundance at 2 h following resistance exercise. Furthermore, intramuscular production of TXA_2_ (measured by local TXB_2_ and 12(S)‐HHTrE concentrations) increased at 2 h postexercise. This finding within the exercised musculature is consistent with prior reports of transiently increased systemic TXB_2_ concentrations following both acute maximal aerobic (Laustiola et al. 1984) and resistance exercise (Markworth et al. 2013). COX enzymes are also able to metabolize linoleic acid to form hydroxyoctadecadienoic acids (HODEs), which function stimulate the maturation of monocytes to form macrophages. Previous research has identified an increase in plasma in 9‐ and 13‐HODE following 75 km of cycling (Nieman et al. 2018), however this study is the first to detect an increase in 9‐ and 13‐ HODEs in skeletal muscle tissue following acute resistance exercise.

Another major metabolic pathway leading to the formation of lipid species involved in the regulation of inflammation is the 5‐lipoxygenase pathway. 5‐LOX‐derived LTB_4_ is increased in human blood serum following acute resistance exercise (Markworth et al. 2013) and high speed running (Hilberg et al. 2005). LTB_4_ is a potent neutrophil chemoattractant and a powerful stimulator of vasoconstriction and blood vessel permeability (Massoumi and Sjölander 2007). Expression of 5‐LOX is essentially limited to bone‐marrow‐derived cells including inflammatory neutrophils and monocytes/macrophages (Rouzer et al. 1989). It is therefore not surprising that in this study LTB_4_ was very lowly expressed in skeletal muscle tissue prior to exercise. At 2 h postexercise, LTB_4_ was present at detectable concentrations in muscle biopsies from the majority of subjects, indicative of a potential increase from resting levels. Consistently, downstream derivatives of LTB_4_, including 12‐Oxo LTB_4_ and 20‐COOH LTB_4,_ increased above resting levels at 2 h postexercise. A less well‐described branch of the 5‐LOX pathway involves the metabolism of 22‐carbon n‐3 PUFA DHA to form mono‐hydroxylated fatty acids 4‐ and 7‐HDoHE. Both of these fatty acids were detected in resting skeletal muscle tissue and increased above basal levels at 2 h postexercise. On the other hand, we observed no change in 4‐ or 7‐HDoHE during recovery from resistance exercise in human blood serum samples previously (Markworth et al. 2013).

In addition to 5‐LOX, metabolites of the human platelet type 12‐LOX enzyme 12‐HETE and its downstream derivate tetranor 12‐HETE were also elevated within muscle postexercise. Both metabolites are pro‐inflammatory in nature and act transcellularly to modify the responsiveness of neutrophils to other chemotactic factors (Reynaud and Pace‐Asciak 1997). We previously observed a similar increase in 12‐HETE and downstream tetranor 12‐HETE in human blood serum samples during recovery from resistance exercise (Markworth et al. 2013). Interestingly 12‐LOX‐expressing platelets are implicated in the transcellular biosynthesis of pro‐resolution LX mediators through interactions with 5‐LOX expressing PMNs. This pathway involves leukocyte–platelet interactions during which the 5‐LOX‐derived leukotriene intermediate LTA_4_ is taken up by 12‐LOX expressing platelets for subsequent conversion to LXA_4_ (Serhan and Sheppard 1990; Romano et al. 1993). Further, the 15‐LOX pathway is implicated as a second endogenous route of LX biosynthesis species. 15‐LOX is highly expressed in alternatively activated macrophages and epithelial cells. The secretion of the 15‐LOX product, 15‐HETE, can be taken up by 5‐LOX expressing cells and converted to LXA_4_ and LXB_4_ (Serhan 1989)_._ In this study, we observed a trend toward an increase in 15‐HETE 2 h postexercise, which supports our prior observation in human blood serum samples (Markworth et al. 2013). The simultaneous induction of the primary products of both the 5‐LOX/12‐LOX and 15‐LOX/5‐LOX pathways within skeletal muscle during postexercise recovery is presumably a permissive environment for local LX biosynthesis. We were, however, unable to detect LXA_4_ or LXB_4_ themselves within the muscle biopsy homogenates analyzed here. Nevertheless, these local changes within the exercised musculature suggests that skeletal muscle tissue may potentially contribute to the previously reported systemic lipoxin response to resistance exercise following their release from exercised myofibers (Markworth et al. 2013).

The CYP pathway is a third and less well‐characterized branch of the AA metabolic pathway. The increase in epoxyeicosatrienoic acid regioisomer 5‐6‐EpETrE and dihydroxyeicosatrienoic acids 11,12‐ and 14,15‐DiHETrE in skeletal muscle supports observations made in serum samples when measured during the early stages of postexercise inflammation (Markworth et al. 2013). The physiological function of these derivatives remains unexplored in skeletal muscle tissue. However, in vascular smooth muscle and endothelium cells they play anti‐inflammatory roles through in the inhibition of prostaglandin and cytokine induced inflammatory responses (Spector et al. 2004). CYP enzymes also metabolize linoleic acid via epoxidation to form epoxy*‐*octadecanoic acids (EpOMEs). EpOMEs are rapidly hydrolyzed by the sEH enzyme to form corresponding dihydroxy*‐*octadecanoic acids (DiHOMEs) (Konkel and Schunck 2011). EpOMEs and DiHOMEs are leukotoxins that play a role in the suppression of neutrophil respiratory burst activity, vasodilation and cellular apoptosis (Thompson and Hammock 2007; Nieman et al. 2014). A cycling‐based intervention comprising of a 75 km time trial had no effect on 9‐10,DiHOME 1.5 h and 21 h postexercise in plasma samples of competitive road cyclists (Nieman et al. 2014). Alternatively, a bout of acute resistance exercise triggered an increase in 9(10)‐EpOME and 9‐10,DiHOME in serum samples (Markworth et al. 2013). Results from this study showed an increase in 9,10‐DiHOME and 12,13‐DiHOME, suggesting that discrepancies in the previous literature may be due to the differences in the type of exercise performed and the training status of the subjects.

Collectively, these findings demonstrate an increase in bioactive lipid‐derived mediators of the COX, LOX, and CYP pathways during postexercise muscle recovery. The concept of a biologically active inflammatory resolution program governed by lipid derivatives was first proposed in a TNF‐ɑ‐stimulated model of acute inflammation in the murine air pouch (Levy et al. 2001). Within this model, early formation of LTB_4_ and PGE_2_ at the onset of inflammation was succeeded by a class‐switching of eicosanoids to LXA_4._ During this process, interactions between inflammatory and host tissue cells enabled the biosynthesis of resolution mediators (Levy et al. 2001). Alternatively, in a model of zymosan‐A stimulated murine peritonitis, the onset of inflammation was characterized by a concomitant increase in LTB_4_ and LXA_4_ followed by a late appearance of PGE_2_ at the onset of resolution (Bannenberg et al. 2005). These findings demonstrate that the temporal regulation of lipids and their role in inflammation is likely cell‐type and stimulus specific. Recent work from our group profiled the human lipid response to acute resistance exercise in serum samples (Markworth et al. 2013). This study showed an increase in key prostaglandin, leukotriene, lipoxin, and resolvin species during the early stages of acute inflammation (1–3 h), followed by an increase in 15‐LOX derivatives and some prostaglandin metabolites (6‐keto‐PGF_1ɑ_ and 13,14dh‐15kPGE_2_) 24 h postexercise (Markworth et al. 2013). The present finding of increased intramuscular abundance of bioactive lipid mediators at 2 h postexercise is overall consistent with findings from serum samples (Markworth et al. 2013), suggesting that muscle may be a major source of blood lipid mediators. Interestingly lipid species that require transcellular interactions, including lipoxins, resolvins, and protectins, were either undetected, or very lowly expressed in skeletal muscle tissue, disproving the original hypothesis predicting a delayed increase in SPMs coincident with the resolution of acute inflammation. These lipids were detectable previously in human serum samples during postexercise recovery and play a vital role in the active resolution of acute inflammation (Markworth et al. 2013).

## Conclusion

This is the first study to characterize the lipid mediator profile of human skeletal muscle tissue at rest and following acute resistance exercise. We identified an increase in lipids autocoids derived from the COX, LOX, and CYP pathways. Peak induction of AA‐derived classical proinflammatory prostaglandins (TXB_2_, PGE_2_ and PGF_2ɑ_) and leukotrienes (LTB_4,_ 12‐Oxo LTB_4_ and 20‐COOH LTB_4_) occurred at 2 h postexercise. Further various derivatives of the 5‐LOX (5‐HETE, 4‐HDoHE, and 7‐HDoHE), 12‐LOX (12‐HETE, tetranor 12‐HETE, and 14‐HDoHE) and 15‐LOX (15‐HETE) pathways were identified in abundance in skeletal muscle tissue at 2 h postexercise and may resemble transient cellular intermediates for the formation of pro‐resolution lipoxin and resolvin species. In alternative models of acute inflammation, these lipids are involved in coordinating a biologically active inflammatory resolution program that is mechanistically linked to tissue healing. This study was limited in that the analysis was performed during the first 24 h of postexercise recovery. Later time points may provide insight into an ongoing cascade of complex lipidomic alterations (Serhan et al. 2007, 2008; Ryan and Godson 2010). This study represents a descriptive analysis of the skeletal muscle lipid response to acute resistance exercise. Further mechanistic research exploring the physiological significance and function of these lipids, both locally within skeletal muscle tissue and following their systemic release, will be useful in further characterizing the significance of the intramuscular inflammatory response in postexercise muscle recovery.

## Conflict of Interest

This research was conducted in the absence of any commercial or financial relationships that could be construed as a potential conflict of interest.

## Supporting information




**Table S1.** Complete list of lipid mediators detected in human skeletal muscle tissue. Values are mean ± SEM of tissue lipid mediators concentration (ng/g).
